# How Managers’ Job Crafting Reduces Turnover Intention: The Mediating Roles of Role Ambiguity and Emotional Exhaustion

**DOI:** 10.3390/ijerph17113972

**Published:** 2020-06-03

**Authors:** Yuhyung Shin, Won-Moo Hur, Kyungdo Park, Hansol Hwang

**Affiliations:** 1 School of Business, Hanyang University, 17 Haengdang-dong, Seongdong-gu, Seoul 133-791, Korea; yuhyung@hanyang.ac.kr (Y.S.); hansolhwang@hanyang.ac.kr (H.H.); 2 College of Business Administration, Inha University, 100 Inha-ro, Michuhol-gu, Incheon 22212, Korea; 3 Sogang Business School, Sogang University, Seoul 04107, Korea; kyungdo@sogang.ac.kr

**Keywords:** manager job crafting, role ambiguity, emotional exhaustion, turnover intention

## Abstract

Despite the increasing body of research on job crafting, the relationship between managers’ job crafting and their turnover intention, as well as its intermediary mechanisms, has received relatively little attention from researchers. This study examined how managers’ job crafting negatively affected their turnover intention, focusing on role ambiguity and emotional exhaustion as underlying mediators. Data were collected from 235 store managers in South Korean food franchises. All study hypotheses were supported by regression-based path modeling. Controlling for role conflict and role ambiguity, we found a negative relationship between job crafting and role ambiguity, a positive relationship between role ambiguity and emotional exhaustion, and a positive relationship between emotional exhaustion and turnover intention. Our mediation analyses further revealed that controlling for role conflict and role overload, role ambiguity and emotional exhaustion partially and sequentially mediated the relationship between managers’ job crafting and their turnover intention. These findings have several implications for theory and practice.

## 1. Introduction

It is needless to say that managers play a pivotal role in creating and maintaining enhanced organizational performance. Given that organizational effectiveness depends on managers’ expertise, decision-making abilities, and leadership, retaining talented managers is a key to organizational success [1]. Therefore, the investigation of factors affecting managers’ turnover intention (i.e., the intention to leave the organization) constitutes an important subject of research [2,3]. Of the various factors predicting turnover intention, job characteristics (i.e., task variety, task identity, feedback, autonomy, and task significance) have been identified as the antecedents of managers’ turnover intention [4]. More precisely, when provided with high levels of task variety, task identity, feedback, autonomy, and task significance, managers manifest less turnover intention [4].

Prior research on managers’ turnover intention, however, has two major limitations. First, while this line of research suggests that the motivating characteristics of a job can lead to a decrease in managers’ turnover intention [4], how to make their job motivating and meaningful has rarely been explored in the literature on managerial turnover. To fill this gap, in the present study, we isolate job crafting as a job-redesign technique that renders managers’ jobs motivating and meaningful, which, in turn, reduces their turnover intention. Job crafting refers to physical or cognitive changes individuals make in their tasks [5]. Distinct from traditional top-down job designs, job crafting is characterized by job holders’ own initiatives to alter the content and meaning of their job, as well as the quality of their relationship with others in the workplace. Although the majority of job crafting research has focused on job crafting by employees, mounting research has demonstrated that managers’ job crafting is positively associated with their job satisfaction and promotions (e.g., [6]) and unit-level performance (e.g., [7]). Given the positive effect of job crafting on managers’ attitudinal and performance outcomes, we propose job crafting as a crucial job-redesign activity that contributes to reduced managers’ turnover intention.

The other limitation of prior research on managers’ turnover intention lies in neglecting mechanisms that mediate between job characteristics and managers’ turnover intention. Scholars have noted that there is little research on the intermediary processes through which job characteristics are associated with turnover intention [4,8]. In response to this call for research, we identify role ambiguity and emotional exhaustion as mechanisms underlying the relationship between job crafting and managers’ turnover intention. Drawing on the job demands-resources (JD-R) model [9], we posit that job crafting reduces managers’ turnover intention by alleviating hindering job demands (i.e., role ambiguity), and the resulting emotional exhaustion. As such, the purpose of our study is to propose and test the mediating effect of role ambiguity and emotional exhaustion on the relationship between managers’ job crafting and turnover intention. We further postulate that there is a serial relationship between job crafting, role ambiguity, emotional exhaustion, and turnover intention even after controlling for job-stress (i.e., role conflict and role overload) and demographic covariates. Because there are a number of job and personal characteristics that affect emotional exhaustion and turnover intention in the JD-R model, the proposed relationships can be rigorously estimated when other important covariates are controlled for.

**Figure 1:**
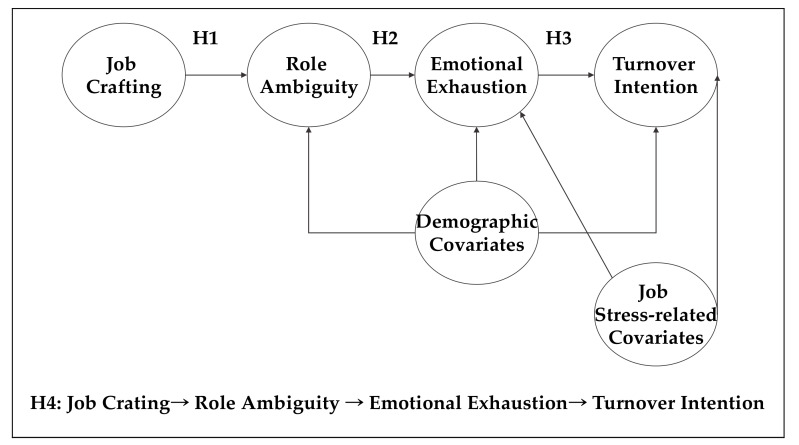
Proposed research model and research hypotheses.

Our research is expected to make three contributions to the job crafting literature. First, despite a vast amount of research on employees’ job crafting and its ramifications (for a meta-analytic review, see [10]), the role that job crafting plays in managerial outcomes has rarely been explored. Our research is one of the first to examine the role of job crafting in predicting managers’ turnover intention. Second, investigating the link between job crafting and managers’ turnover intention is of practical importance. Considering the tangible costs resulting from the attrition of talented managers [2,11,12], inventing ways of preventing and reducing managerial turnover is a conundrum that many organizations are facing [3,4]. By suggesting job crafting as a means to reduce managers’ turnover intention, our research offers practical solutions to managerial turnover. Third, despite the finding that motivating jobs can decrease managers’ turnover intention, the underlying mechanisms through which job crafting reduces managers’ turnover intention remain unclear. Adopting the JD-R model as a theoretical framework, our research highlights the importance of role ambiguity and emotional exhaustion as central mediating mechanisms, which, in turn, advances our understanding of the relationship between job crafting and turnover intention. The proposed research model is depicted in Figure 1.

## 2. Theoretical Background and Hypotheses

### 2.1. The JD-R model

The basic assumption of the JD-R model is that working conditions elicit two distinct psychological responses: work engagement and burnout [9]. While work engagement is defined as “a positive, fulfilling affective-motivational state of work-related well-being that is characterized by vigor, dedication, and absorption” [13] (p. 187), burnout is a work-related stress syndrome. Given that emotional exhaustion is a symptomatic manifestation of burnout [14,15], in line with prior work grounded in the JD-R framework [2], we focus on emotional exhaustion as a key dimension of burnout. Emotional exhaustion refers to the depletion of emotional resources stemming from the exposure to certain job demands [14,16,17]. In the JD-R framework, job resources have a positive impact on employee performance and well-being by enhancing work engagement, whereas job demands undermine performance and well-being by incurring emotional exhaustion. Job resources are physical, psychological, social, or organizational aspects of a job that are functional in attaining work goals [17]. In contrast, job demands are physical, psychological, social, or organizational aspects of a job that are demanding, and therefore, can result in exhaustion [17]. Job demands are further categorized into challenging and hindering, depending on whether they are beneficial (e.g., new roles and increased responsibility) or detrimental (e.g., job insecurity, role ambiguity, and interpersonal conflicts) to goal achievement, personal growth, and well-being [18]. According to the JD-R model, as hindering job demands are emotionally taxing, they impede the individuals’ goal attainment by draining their emotional resources.

In the present study, we argue that role ambiguity is a key hindering job demand to managers. Role ambiguity is defined as the extent to which a person is uncertain about what is required for the adequate performance of his/her role [19,20,21,22]. Role ambiguity is of particular importance to managers, who play a boundary-spanning role in organizations [23,24,25]. For instance, middle managers serve as a communication channel between upper management and employees, and often need to fulfill different demands between these two parties (e.g., upper management’s expectations for profits and efficiency vs. employees’ expectations for high salary and decent fringe benefits). Likewise, branch or store managers need to accommodate different demands from the company’s higher management and customers (e.g., controlling costs vs. providing high-quality products or services). As such, managers interacting with multiple role senders are unlikely to define their role expectations clearly [26]. Unless managers have a clear sense of the required behavior and goals, they are likely to experience stress and frustration in their role [24,25], which functions as a hindering job demand. Therefore, we identify role ambiguity as a key hindering job demand that is strongly associated with managers’ emotional exhaustion and resulting turnover. From a JD-R lens, job crafting encompasses activities that increase job resources and challenging job demands and decrease hindering job demands [27]. Based on this theorizing, we argue that job crafting decreases hindering job demands (i.e., role ambiguity), which, in turn, reduces emotional exhaustion and turnover intention. The proposed relationships are explained in detail in the following sections.

### 2.2. Relationship between Managers’ Job Crafting and Role Ambiguity

JD-R theorists initially defined job crafting as activities that increase social job resources (e.g., social support, feedback, and mentoring), structural job resources (e.g., job autonomy and opportunities for growth and development), and challenging job demands (e.g., taking on new assignments), and decrease hindering job demands (e.g., job insecurity) [27]. Prior research has focused on the former three activities because increasing job resources and challenging job demands have stronger motivational power than the reduction in hindering job demands (e.g., [7,28,29,30,31,32,33]). Zeijen et al. [33] claimed that when examining the motivational process involved in job crafting, it is reasonable to exclude decreasing hindering job demands. Therefore, consistent with previous job crafting research and Cenciotti et al.’s [6] conceptualization of manager job crafting, we define manager job crafting as the extent to which a manager increases social and structural job resources and challenging job demands.

We predict that managers’ job crafting is negatively associated with role ambiguity through the following mechanisms. First, managers’ efforts to increase social job resources can reduce role ambiguity. Examples of social job resources are feedback and coaching from others in work relationships. Feedback enables managers to clarify performance expectations from different stakeholders [34]. Furthermore, performance feedback assists managers to understand their roles [35] and reduce their role ambiguity [26]. Therefore, managers who actively seek feedback from multiple stakeholders come to have clear perceptions of roles and expectations imposed on them, thereby experiencing less role ambiguity. Coaching and mentoring from senior managers can also help managers better understand managerial roles and better deal with conflicting demands from different stakeholders. Thus, seeking coaching and mentoring from more experienced managers should be associated with decreased role ambiguity.

Second, increasing structural job resources pertains to developing and utilizing managers’ competencies and enhancing job autonomy [27]. When managers develop their knowledge, skills, and abilities through job crafting, these competencies match their role requirements [7]. Moreover, the acquisition of knowledge, skills, and abilities required for their jobs enables managers to realize their goals [36] and align their actual roles with expected roles [37], which, in turn, results in low role ambiguity. In addition, when managers are allowed autonomy in their job, they can set their own performance goals and determine the contents, methods, and schedule of their work, thereby experiencing less ambiguity in their roles [38,39]. Increasing job autonomy also diminishes managers’ role ambiguity through the integration of different tasks into their roles [40]. Indeed, job autonomy has been found to be negatively related to role ambiguity (e.g., [39]).

Third, increasing challenge job demands involves expanding roles, responsibility, tasks, or assignments. However, this means not merely increasing the number of roles and assignments, but taking on new roles or assignments that potentially contribute to one’s own advancement and development [31]. As these new roles and assignments are aligned with managers’ own goals in job crafting contexts, managers become better capable of prioritizing among the existing and newly-added roles, and therefore, experience less role ambiguity. Taken together, managers who increase social and structural job resources and challenging job demands are anticipated to exhibit a low level of role ambiguity. Hence, it is hypothesized that:

**Hypothesis** **1.**  Managers’ job crafting is negatively related to their role ambiguity.

### 2.3. Relationship between Managers’ Role Ambiguity and Emotional Exhaustion

The job crafting literature grounded in the JD-R model maintains that job crafting results in the reduction in hindering job demands, which, in turn, is associated with decreased emotional exhaustion [41]. This is because the reduction in role ambiguity depletes fewer resources. According to the JD-R model, prolonged exposure to hindering job demands drains individuals’ emotional resources and energy [17]. As a result, role ambiguity is expected to have a positive relationship with emotional exhaustion. This relationship has been empirically supported across different samples and contexts (e.g., [23,42,43,44,45,46]). In particular, the detrimental effect of role ambiguity is more severe among managers who act as boundary spanners [23]. Because managers need to accommodate and coordinate different demands from multiple stakeholders, the lack of clarity in roles imposed by different stakeholders is stressful to managers [25]. In addition, when facing role ambiguity, managers need to expend cognitive and psychological resources to resolve this ambiguity, which leads to emotional exhaustion. Role theorists argue that role ambiguity creates confusion, tension, and anxiety, resulting from not knowing what to do [19,47,48]. Such frustration and anxiety are primary causes of emotional exhaustion [44]. As a result, managers with ambiguous roles are likely to exhaust their emotional resources. Likewise, uncertainty about job duties and responsibilities incurs negative experiences such as burnout [49,50,51,52]. Therefore, we predict the following relationship:

**Hypothesis** **2.**  Managers’ role ambiguity is positively related to their emotional exhaustion.

### 2.4. Relationship between Managers’ Emotional and Turnover Intention

Emotional exhaustion is known as a key mediator linking role stress and turnover intention [53]. The positive link between emotional exhaustion and turnover intention has been well documented (e.g., [2,36,53,54,55,56]). This relationship can be explained by resources theories such as the JD-R model and conservation of resources (COR) theory. In the JD-R framework, mental fatigue arising from hindering job demands exerts a chronic adverse effect on individuals’ health and well-being [17], which causes them to withdraw from work. Central to COR theory is that individuals strive to obtain, retain, and protect valued resources [57]. COR theory holds that stress occurs when individuals are threatened with resources loss, and that exhausted individuals often use withdrawal coping mechanisms to prevent further loss of resources and psychological costs of exhaustion [57,58,59,60]. As emotionally exhausted managers feel reluctant to expend additional resources in their job, they diminish their commitment to the organization and seek an alternative job or employer [61]. This line of reasoning leads to the following hypothesis:

**Hypothesis** **3.**  Managers’ emotional exhaustion is positively related to their turnover intention.

### 2.5. Mediation of Role Ambiguity and Emotional Exhaustion

Synthesizing the relationships proposed in the previous sections, it is reasonable to put forth a sequential mediation model in which managers’ job crafting is negatively associated with turnover intention through reduced role ambiguity and emotional exhaustion. Although virtually no research has tested this mediation, similar relationships have been revealed in the managerial turnover literature. For instance, Knudsen et al. [2], in their study of leaders’ turnover intention, found that job demands are positively related to emotional exhaustion, which, in turn, is positively associated with turnover intention. Consistent with this finding, Agarwal and Gupta [4] reported that motivating job characteristics decrease managers’ turnover intention through diminished work engagement. These findings suggest that increasing job resources and decreasing hindering job demands reduce managers’ turnover intention by alleviating their emotional exhaustion.

The overall structure of our mediation model is embedded in the JD-R model. The JD-R model asserts that hindering job demands are a major stressor that incurs emotional exhaustion and resulting turnover intention. This model further contends that job crafting is an effective intervention that prevents such a negative process [27]. Job crafting reduces managers’ role ambiguity by increasing structural and social job resources and challenging job demands. Seeking these helpful resources and demands leads managers to better understand their roles by adjusting the scope and content of their tasks to the role requirements and acquiring knowledge, skills, and abilities necessary for their positions. Furthermore, according to the JD-R model, job crafting serves to clarify managers’ roles by balancing their job demands and resources with their abilities, preferences, and needs [62,63,64]. Similarly, from a person–environment fit perspective, job crafting enhances the match between job characteristics and personal needs and abilities [65,66,67,68]. Crafting jobs according to personal needs and abilities increases the correspondence between work content and work identity, thereby making managers feel less frustrated with their roles [67]. As such, low role ambiguity resulting from job crafting does not exhaust managers’ emotional resources, which decreases their turnover intention. Based on this reasoning, we formulate the following mediation hypothesis:

**Hypothesis** **4.**  Role ambiguity and emotional exhaustion sequentially mediate the relationship between managers’ job crafting and turnover intention.

## 3. Method

### 3.1. Sample and Procedure

The sample for the present study was drawn from South Korean food franchises. We randomly selected 600 franchise stores using the list of food franchises located in the southeast region of South Korea. Of these 600 stores (e.g., casual dining restaurants, fast food franchisees, coffee shops/bakeries), 235 stores of 23 franchises consented to participate (response rate = 39.2%), 88% of which belonged to South Korean domestic franchises, and the rest were multinational franchise stores. We compared the characteristics (i.e., store type and size) of the participating stores and non-participating ones, and found that the two groups were not significantly different in their characteristics. On-site research assistants administered paper-and-pencil surveys to store managers. In each store, there was only one manager who was in charge of sales management, human resource management, and customer service. Store managers received a gift card of USD $8 for study participation. Thirty-four per cent of store managers were male. The average age and years in the current store were 32.24 (SD = 8.04) years and 2.79 years (SD = 2.87), respectively. The average size of the participating stores was 10.22 (SD = 3.52) employees, ranging from 6 to 20 employees.

Our research was conducted in accordance with the 1964 Declaration of Helsinki and its later amendments or comparable ethical standards. Our data collection procedure abided by the ethical standards of the institutional and national research committees. Prior to survey administration, respondents were assured that their responses were anonymous and confidential.

### 3.2. Measures

As the original survey items were developed in English, we back-translated them using Brislin’s [69] procedure. All survey items are presented in Table 1. Responses to these items were made on five-point Likert-type scales.

#### 3.2.1. Job Crafting

In line with Cenciotti et al.’s [6] study, we assessed managers’ job crafting based on Tims et al.’s [27] three sub-dimensions of job crafting (α = 0.76): increasing structural job resources (five items, α = 0.89), increasing social job resources (five items, α = 0.90), and increasing challenging job demands (four items, α = 0.84). Following Cenciotti et al.’s [6] practice, we aggregated managers’ scores on these three sub-dimensions to yield a job crafting measure.

#### 3.2.2. Role Ambiguity

To measure managers’ role ambiguity, we used three items (α = 0.83) from Babakus, Yavas, and Ashill’s [70] scale.

#### 3.2.3. Emotional Exhaustion

Emotional exhaustion was assessed using nine items (α = 0.80) of the Burnout Inventory developed by Maslach and Jackson [15]. When the ratio of sample size to number of free parameters is low, item parceling is appropriate for ensuring the validity of model estimation [71]. Adopting the item-to-construct balance approach [72], we created three parcels of emotional exhaustion using random parceling [73]. Parcel 1 consisted of three items (i.e., “I feel emotionally drained/exhausted from work”, “I feel frustrated with my jobs”, and “I feel like I am working too hard on my jobs”). Parcel 2 was composed of three items (i.e., “I feel fatigued when I get up in the morning and have to face another day on the job”, “Working with people all day is really a strain for me”, and “I feel used up at the end of the workday”). Parcel 3 consisted of three items (i.e., “I feel burned out from my work”, “Working directly with people puts too much stress on me”, and “I feel like I am at the end of my rope”).

#### 3.2.4. Turnover Intention

Managers’ turnover intention was measured with four items (α = 0.84) derived from Babakus et al.’s [70] scale.

#### 3.2.5. Control Variables 

Due to their confounding effects on role ambiguity [70], emotional exhaustion [74], and turnover intention [75], we controlled for managers’ gender, age, and job tenure (i.e., years in the current store) in all subsequent analyses. First, according to meta-analytic findings [76], female employees feel more emotionally exhausted than male employees, which suggests a need to control for gender (0 = female, 1 = male). Second, drawing on the meta-analytic finding indicating significant associations between age and emotional exhaustion and between job tenure and emotional exhaustion, we controlled for managers’ age and job tenure [77]. Third, meta-analytic findings [78] also suggest a significant relationship between demographic characteristics (e.g., gender, age, and job tenure) and turnover intention. Thus, it was necessary to control for these demographic characteristics. Lastly, consistent with Ambrose et al.’s [23] findings and meta-analytic findings [79], we controlled for other forms of role stress (i.e., role conflict and role overload) to partial out their potential effect on emotional exhaustion and turnover intention. Six items from Babakus et al.’s [70] scale were used as measures of role conflict (α = 0.80) and role overload (α = 0.80) (see Table 1).

### 3.3. Analytic Strategy

As our data were nested within 23 franchises, we assessed between-franchise variability to determine whether it was necessary to specify a multilevel path model. The intraclass correlation coefficient (ICC1) values for role ambiguity (0.08), role conflict (0.05), role overload (0.07), emotional exhaustion (0.04), and turnover intention (0.03) fell into or below the low category (i.e., low = 0.05~0.09; moderate = 0.10~0.14; high = above 0.15), suggesting that managers’ responses for these variables did not significantly differ across 23 franchises [80]. Therefore, we employed unilevel regression-based path modeling in hypothesis testing [81]. We tested the hypotheses using the M-plus 8.4 software [82]. We also conducted a 95% bias-corrected bootstrapping analysis (*n* = 5000) to test the proposed serial mediation (Hypothesis 4) ([83,84]).

## 4. Results

### 4.1. Tests of Reliability, Validity, and Common Method Variance

Table 2 reports the descriptive statistics and inter-correlations of the study variables. We carried out confirmatory factor analyses (CFAs) to assess the convergent and discriminant validity of our measures. Table 2 shows that all average variance extracted (AVE) values were greater than the squared correlation between the construct and any of the other variables [85]. In addition, as illustrated in Table 3, the hypothesized six-factor model (i.e., job crafting, role ambiguity, role conflict, role overload, emotional exhaustion, and turnover intention) fitted the data in an absolute sense (χ^2^(387) = 713.44; *p* < 0.05, confirmatory fit index [CFI] = 0.92, Tucker–Lewis Index [TLI] = 0.91, root mean square error of approximation [RMSEA] = 0.06, standardized root mean square residual [SRMR] = 0.06), and exhibited a significantly better fit than did any other alternative measurement model. These findings confirm the discriminant validity of our measures.

Because we used self-reported data, we took several procedures to reduce common method variance (CMV). First, based on Podsakoff, MacKenzie, and Podsakoff’s [86] remedies to minimize CMV, we assured the anonymity and confidentiality of responses and improved the wording of survey items by having two management scholars review the items and correcting any unclear or incomprehensible expressions identified by them. Second, we performed Harman’s one-factor analysis as a statistical remedy for CMV [86]. As depicted in Table 3, the one-factor model (χ^2^_(409)_ = 2731.41; *p* < 0.05, CFI = 0.40, TLI = 0.36, RMSEA = 0.16, SRMR = 0.15) demonstrated a worse fit than our measurement model (△χ^2^(22) = 2027.97; *p* < 0.01). Third, we estimated an additional latent common method factor (LCMF), on which each item in the baseline model was allowed to load in addition to loading on its corresponding construct. The LCMF explained 0.85% of the total variance, which was much lower than the median method variance (25%) observed in research using self-reported data [87]. Therefore, it is unlikely that CMV affected the findings of the current study.

### 4.2. Hypotheses Testing

Hypothesis 1 proposed a negative relationship between manager job crafting and role ambiguity. As shown in Table 4, manager job crafting was negatively associated with role ambiguity (*b* = −0.48, *p* < 0.01). In contrast, manager job crafting was not related to role conflict (*b* = 0.11, *p* > 0.05) and role overload (*b* = −0.01, *p* > 0.05). These findings lend support to Hypothesis 1. Hypothesis 2 postulated a positive link between role ambiguity and emotional exhaustion. Controlling for role conflict and role overload, role conflict was positively related to emotional exhaustion (*b* = 0.30, *p* < 0.01), supporting Hypothesis 2. Hypothesis 3 predicted a positive relationship between emotional exhaustion and turnover intention. In support of this hypothesis, emotional exhaustion was positively associated with turnover intention (*b* = 0.43, *p* < 0.01).

**Figure 2:**
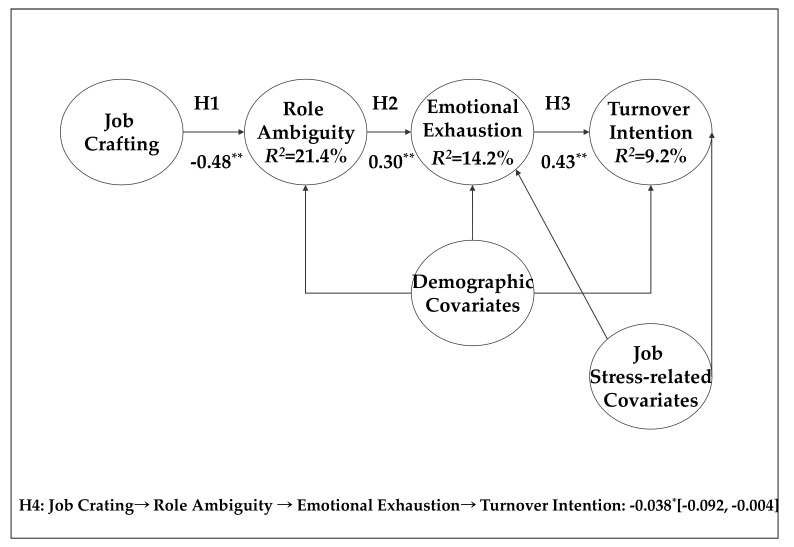
Proposed research model and summary of results. Unstandardized coefficients are reported. For parsimony, path coefficients for demographic and role stress covariates are omitted. * *p* < 0.05, ** *p* < 0.01.

Hypothesis 4 proposed the sequential mediation of role ambiguity and emotional exhaustion in the relationship between manager job crafting and turnover intention. To test this mediation, we assessed the indirect effects, as well as the symmetric and 95% bias corrected bootstrapped confidence intervals for the path estimates (*N* = 5000, [83,84]). The results for the indirect effects are reported in Figure 2. As expected, role ambiguity and emotional exhaustion sequentially mediated the relationship between manager job crafting and turnover intention (*b* = −0.038, 95% CI = [−0.092, −0.004]), thus, supporting Hypothesis 4. Although not hypothesized, we found a significant, negative relationship between job crafting and turnover intention (*b* = −0.352, 95% CI = [−0.570, −0.135]). The magnitude of this relationship was greater than that of the serial mediation effect. The results of hypothesis testing are summarized in Figure 2.

## 5. Discussion

The objective of the present study was to examine a mediating relationship in which managers’ job crafting reduces their turnover intention by decreasing role ambiguity and emotional exhaustion. The results of our mediation analyses supported all hypotheses. As predicted, we found a negative relationship between managers’ job crafting and role ambiguity, a positive relationship between role ambiguity and emotional exhaustion, and a positive relationship between emotional exhaustion and turnover intention. In addition, we found a significant indirect effect of managers’ job crafting on turnover intention through role ambiguity and emotional exhaustion. These effects held after controlling for other forms of role stress (i.e., role conflict and role overload). Our findings have implications for theory and practice.

### 5.1. Theoretical Implications

Compared with the vast amount of research on employee job crafting, knowledge about how job crafting contributes to managerial outcomes is limited, although a few studies have demonstrated that manager job crafting is beneficial to managers’ career success and unit-level performance (e.g., [6,7]). While these studies suggest a positive association between managers’ job crafting and their promotions and unit-level performance, the underlying mechanisms remain a black box. As shown in our research, turnover intention can be a potential mediator between manager job crafting and managerial outcomes. More precisely, managers who craft their jobs experience less turnover intention (through diminished role ambiguity and emotional exhaustion), which, in turn, leads to increased promotions and unit-level performance. Considering the economic value of retaining talented managers, their decreased turnover intention should bring positive consequences to the bottom line of the unit they manage. In this regard, examining turnover intention as an outcome of manager job crafting offers important insights for the job crafting literature. The negative association between manager job crafting and turnover intention is consistent with the job crafting–turnover relationship that has been found at the employee level (for a meta-analytic review, see [10]). Parallel to the relationship that occurs among employees, job crafting serves to decrease managers’ turnover intention, highlighting the significance of job crafting in retaining employees and managers. Based on these findings, job crafting can be considered a useful intervention to prevent and reduce turnover in organizations.

Our study makes a theoretical contribution to the job crafting literature by taking a JD-R approach to job crafting. Job crafting research grounded in the JD-R framework proposes increasing challenging job demands, structural job resources, and social job resources as the three core components of job crafting, which are associated with increased work engagement and decreased emotional exhaustion. Consistent with this line of research, our findings indicated that increasing challenging job demands, structural job resources, and social job resources is associated with decreased turnover intention by reducing role ambiguity and emotional exhaustion. Given that role ambiguity is a major hindering job demand in the JD-R model, the finding that job crafting is negatively related to role ambiguity also endorses the JD-R model. The JD-R model further posits that hindering job demands is a key antecedent of emotional exhaustion, which was supported in our analyses. Furthermore, the positive link between emotional exhaustion and turnover intention is consistent with prior findings from a JD-R lens [2]. As predicted by the JD-R model, emotional exhaustion, by draining managers’ physical and psychological resources, serves to dampen their intention to remain in their current job. As such, by validating the JD-R model through our mediation analyses, our findings suggest that the JD-R model is a pertinent framework for explaining the effect of manager job crafting.

Although we developed our hypotheses on the basis of the JD-R model, the proposed relationships can also be explained by conservation of resources (COR) theory, which posits that individuals strive to preserve and accumulate resources to cope with threats to their well-being [57]. According to COR theory, role ambiguity is harmful to managers’ well-being because it depletes their mental and emotional resources. COR theory further claims that as exhausted people have limited resources, they feel reluctant to invest further resources in their job, which can explain why exhausted managers are likely to quit their job. However, job crafting enables managers to gain resources that protect them from further resource loss, such as role ambiguity and emotional exhaustion [29]. Managers who craft their job can retain mental and emotional resources, which help them effectively cope with role ambiguity. Moreover, job crafting provides managers with resources that protect them from feeling emotionally exhausted [29]. Based on this theorizing, managers who engage in job crafting experience less emotional exhaustion, thereby feeling less inclined to quit their job.

Our findings also contribute to the role stress literature. Of the three forms of role stress, only role ambiguity had a significant negative relationship with job crafting. Furthermore, role ambiguity significantly predicted emotional exhaustion, whereas the relationship between role conflict and emotional exhaustion and the relationship between role overload and emotional exhaustion were not significant at the 0.05 level. This can be explained by the nature of job crafting, which often accompanies the addition of new roles and assignments (i.e., increasing challenging job demands). When roles and assignments are expanded through job crafting, managers are likely to experience role overload and collision between different roles. For this reason, job crafting may not decrease role conflict and role overload. However, as job crafting involves aligning one’s own motivation, needs, and strengths with job requirements, role ambiguity should be diminished when managers craft their jobs. Moreover, role ambiguity is likely to lead to greater depletion of emotional resources than the other two forms of role stress. Clarifying role expectations imposed by stakeholders requires considerable resources (e.g., receiving and interpreting messages and feedback from stakeholders and internalizing their needs and preferences). As a result, role ambiguity drains managers’ cognitive and emotional resources, thereby rendering them emotionally exhausted. In contrast, role conflict and role overload are less taxing in that they can be easily resolved by reducing the number of roles. Thus, by unraveling the differential relationships between different forms of role stress, job crafting, and emotional exhaustion, our research offers a nuanced understanding of role stress.

Of the three demographic control variables, only gender had a significant relationship with job crafting, role ambiguity, and emotional exhaustion. As shown in Table 2, male managers engaged in more job crafting than female managers (*r* = 0.20, *p* < 0.01). While this finding is consistent with Petrou, Demerouti, and Xanthopoulo’s [88] finding that men are more likely to engage in job crafting than women, it is in discord with meta-analytic findings that women craft their jobs slightly more than men [10]. Thus, the relationship between gender and job crafting is inconclusive. However, in line with previous findings [76], we found that female managers displayed higher role ambiguity and emotional exhaustion than male managers (*r* = −0.11, *p* < 0.10 for role ambiguity; *r* = −0.17, *p* < 0.01 for emotional exhaustion). These findings suggest that because male and female managers experience different levels of role ambiguity and emotional exhaustion, they may need different strategies to cope with work stressors.

### 5.2. Practical Implications

The present findings highlight the role of job crafting in attenuating managers’ role ambiguity, emotional exhaustion, and turnover intention. To retain talented managers, we suggest organizational leaders to promote their job crafting. Job crafting research has documented the effectiveness of job crafting interventions (for a meta-analytic review, see [89]). Through these interventions, organizations can assist managers to learn specific job crafting skills (e.g., increasing challenging job demands, increasing structural job resources, and increasing social job resources). Once managers are equipped with these skills, they become adept at crafting their jobs, and can therefore act as role models for their subordinates [90]. The dissemination of job crafting from managers to subordinates can create a job crafting climate within the organization, which, in turn, can enhance collective performance [64].

Given that job crafting is a bottom-up process, any job crafting interventions are not successful without job crafters’ ownership and self-directed efforts. In this regard, it is critical that organizations build an environment that supports managers’ job crafting. Allowing greater autonomy and authority can encourage managers to craft their own jobs. In doing so, organizational leaders are advised to exercise empowering leadership toward middle managers so that they can have discretion and autonomy to modify their jobs. Such endeavors to stimulate manager job crafting are likely to elevate the competitive advantage of the organization by retaining high-performing managers.

### 5.3. Limitations and Directions for Future Research

There are several limitations inherent to our study that suggest directions for future research. First, it should be noted that we relied on single-source, cross-sectional data. As all variables were measured simultaneously, we cannot ascertain the causal relationship between job crafting, role ambiguity, emotional exhaustion, and turnover intention. For instance, managers who intend to quit their job are reluctant to exert much effort in their job, displaying a low level of job crafting. Thus, the reverse causality between job crafting and turnover is plausible, which warrants future longitudinal research. In addition, self-reported data are vulnerable to social desirability and evaluation apprehension biases. Although we found that CMV was not a serious threat in our research, it is desirable to temporally separate the independent variable, mediator, and dependent variable, as well as to use multi-source data [86]. While emotional and attitudinal variables (i.e., role ambiguity, emotional exhaustion, and turnover intention) can be best assessed by the focal individual, use of other-rated job crafting can minimize rater biases associated with job crafting.

Second, in our data analyses, we aggregated the three sub-dimensions of job crafting, which is in line with the common practice of measuring managers’ job crafting (e.g., [6,7]). However, there is evidence that the three sub-dimensions reflect distinct aspects of job crafting [27]. Grounded in this theorizing, there may be a specific sub-dimension that has a greater impact on managers’ role ambiguity. For example, of the three sub-dimensions of job crafting, increasing social job resources is likely to decrease role ambiguity by providing managers with feedback that is helpful in clarifying their role expectations. However, increasing challenging job resources could lead to potential role conflict or role ambiguity by expanding the number of tasks or assignments [26]. Thus, increasing social job resources may be more important to the reduction in role ambiguity than increasing challenging job resources. For a more refined understanding of the relationship between manager job crafting and role ambiguity, future research needs to explore the differential roles of the sub-dimensions of job crafting in alleviating role ambiguity and turnover intention.

Third, while we have focused on the mediating relationship between manager job crafting, role ambiguity, emotional exhaustion, and turnover intention in this study, there may be boundary conditions that strengthen or weaken this relationship. Organizational systems or contexts may function as such boundary conditions. More precisely, it is likely that the negative effect of manager job crafting on turnover intention is more pronounced when there are effective organizational reward systems or when managers perceive a high level of organizational support. Because turnover intention is strongly affected by organizational policies and practices, we encourage future researchers to examine the cross-level moderation of organizational factors on the relationship between manager job crafting and turnover intention. Additionally, there are personal characteristics that may interact with job crafting. According to the JD-R proposition suggesting that personal resources boost the positive effect of job resources and buffer the negative effect of job demands [91], individuals’ motivation or personality can amplify the positive effect of job crafting. For instance, the negative effect of job crafting on turnover intention is likely to be stronger for managers with high conscientiousness and emotional stability. We suggest the interplay between managers’ personal resources and job crafting on turnover intention as a future research topic.

Lastly, our sample was limited to only food franchise managers. Thus, the generalizability of the present findings to overall managers is unknown. The National Restaurant Association [92] reported that the employee turnover rate in the restaurants and accommodations sector was 75% in 2018, which is much higher than turnover rates in other retail sectors. Furthermore, 35% of restaurant managers quit in their first year on the job [93]. Because restaurant managers are faced with work stressors such as dealing with customers’ complaints, attaining sales goals, maintaining food and service quality as well as the hygiene of the restaurant, they tend to experience a high degree of job burnout [94]. For this reason, the significant effect of emotional exhaustion on turnover intention found in our research may not be observed in other managerial settings. We therefore suggest future research to validate the present findings on managers in different occupations and industries.

## 6. Conclusions

Despite a large body of research on employee job crafting, very little is known about the role of manager job crafting in managerial outcomes. Considering the potential costs associated with managerial turnover, it is imperative to unravel the relationship between manager job crafting and turnover, as well as its intermediary processes. By proposing job crafting as the antecedent of managerial turnover and demonstrating the sequential mediation of role ambiguity and emotional exhaustion, our research adds to the extant knowledge on job crafting and offers practical implications for managerial turnover. Future investigations into the boundary conditions affecting the job crafting–turnover intention relationship can further insights gained from our research.

## Figures and Tables

**Table 1:** CFA Results of Measurement Items.

Construct	Measurement Items	λ	
Increasing structural job resources	I try to develop my capabilities.	0.81	
	I try to develop myself professionally.	0.80	
	I try to learn new things at work.	0.81	
	I make sure that I use my capacities to the fullest.	0.81	
	I decide on my own how I do things.	0.71	
Increasing social job resources	I ask my supervisor to coach me.	0.74	
	I ask whether my supervisor is satisfied with my work.	0.84	
	I look to my supervisor for inspiration.	0.79	
	I ask others for feedback on my job performance.	0.85	
	I ask colleagues for advice.	0.80	
Increasing challenging job demands	When an interesting project comes along, I offer myself proactively as project co-worker.	0.71	
	If there are new developments, I am one of the first to learn about them and try them out.	0.82	
	When there is not much to do at work, I see it as a chance to start new projects	0.76	
	I try to make my work more challenging by examining the underlying relationships between aspects of my job.	0.74	
Job crafting	Increasing structural job resources	0.75	
	Increasing social job resources	0.71	
	Increasing challenging job demands	0.83	
Role conflict	I find myself trying to meet conflicting demands of various departments.	0.79	
	I have to deal with or satisfy too many different people.	0.75	
	I sometimes have to bend a rule or policy in order to carry out my job.	0.71	
Role overload	I am not given enough time to do what is expected of me on the job. ^®^	0.60	
	The performance standards on my job are too high	0.88	
	It often seems like I have too much work for one person to do.	0.81	
Role ambiguity	Clear goals/objectives exist for my job. ^®^	0.84	
	I know exactly what is expected of me. ^®^	0.82	
	I know how my performance is going to be evaluated. ^®^	0.72	
Emotional exhaustion	Parcel 1	0.83	
	Parcel 2	0.81	
	Parcel 3	0.84	
Turnover intention	I will probably be looking for another job soon.	0.83	
	I often think about quitting.	0.84	
	I will quit this job sometime in the next year.	0.77	
	It would not take too much to make me resign from this store	0.61	
χ^2^_(387)_ = 713.44; *p* < 0.05, CFI = 0.92, TLI = 0.91, RMSEA = 0.06, SRMR = 0.06			

Notes: All items measured on a scale ranging from 1 “strongly disagree” to 5 “strongly agree.” All factor loadings were significant (*p* < 0.01). ^®^ indicates reverse-coded items. CFA = confirmatory factor analysis; CFI = comparative fit index; TLI = Tucker–Lewis Index; RMSEA = root mean square error of approximation; SRMR = standardized root mean square residual.

**Table 2:** Means, standard deviations, and correlations.

	Mean	SD	A	CR	1	2	3	4	5	6	7	8	9
1. Gender	0.34	0.47	-	-	-								
2. Age	32.24	8.04	-	-	−0.11 ^†^	-							
3. Job tenure	2.79	2.87	-	-	0.05	0.29 **	-						
4. Job crafting	3.68	0.52	0.76	0.81	0.20 **	−0.03	−0.03	0.59					
5. Role conflict	2.30	0.79	0.80	0.80	0.05	−0.09	−0.08	0.10	0.56				
6. Role overload	2.86	0.85	0.80	0.81	−0.04	0.10	0.09	−0.02	0.37 **	0.60			
7. Role ambiguity	2.10	0.60	0.83	0.84	−0.11 ^†^	−0.03	−0.04	−0.46 **	−0.13 ^†^	−0.04	0.63		
8. Emotional exhaustion	2.22	0.54	0.80	0.87	−0.17 **	−0.04	−0.01	−0.38 **	0.29 **	0.27 **	0.26 **	0.68	
9. Turnover intention	2.19	0.94	0.84	0.85	−0.09	−0.11	−0.09	−0.36 **	0.11 ^†^	0.21 **	0.19 **	0.48 **	0.59

Notes: Numbers along the diagonal are the average variance extracted. SD = standard deviation; CR = composite reliability. ^†^ *p* < 0.10, ** *p* < 0.01.

**Table 3:** Comparison of measurement models.

Measurement Models	χ^2^	*Df*	△χ^2^	△*df*	CFI	RMSEA
Hypothesized six-factor model	713.44 **	387	-	-	0.92	0.06
Five-factor model: combining role conflict and role overload into a single factor	883.95 **	392	170.55 **	5	0.87	0.07
Four-factor model: combining role ambiguity, role conflict, and role overload into a single factor	1238.01 **	396	524.57 **	9	0.78	0.10
Three-factor model: combining role ambiguity, role conflict, and role overload into one single factor and combining emotional exhaustion and turnover intention into other single factor	1462.14 **	399	748.70 **	12	0.72	0.11
One-factor model	2731.41 **	409	2027.97 **	22	0.40	0.16

Notes: *df* = degree of freedom; CFI = comparative fit index; RMSEA = root mean square error of approximation. ** *p* < 0.01.

**Table 4:** Results of path analysis.

Variables	Role stress Covariates		1st Mediator	2nd Mediator	Dependent Variable
	Role Conflict	Role Overload	Role Ambiguity	Emotional Exhaustion	Turnover Intention
Gender	0.05	−0.06	−0.02	−0.15 *	−0.09
Age	−0.01	0.01	−0.00	−0.00	−0.01
Job tenure	−0.02	0.02	−0.01	−0.00	−0.02
Job crafting	0.11	−0.01	−0.48 **		
Role conflict				0.09 ^†^	
Role overload				0.02	
Role ambiguity				0.30 **	
Emotional exhaustion					0.43 **
*R^2^*	1.8%	1.5%	21.4%	14.2%	9.2%

Notes: Unstandardized coefficients are reported. ^†^ *p* < 0.10, * *p* < 0.05, ** *p* < 0.01.
